# Sparsentan improves glomerular hemodynamics, cell functions, and tissue repair in a mouse model of FSGS

**DOI:** 10.1172/jci.insight.177775

**Published:** 2024-09-03

**Authors:** Georgina Gyarmati, Urvi Nikhil Shroff, Audrey Izuhara, Sachin Deepak, Radko Komers, Patricia W. Bedard, Janos Peti-Peterdi

**Affiliations:** 1 Departments of Physiology and Neuroscience, and Medicine, Zilkha Neurogenetic Institute, University of Southern California, Los Angeles, California, USA.; 2 Travere Therapeutics, San Diego, California, USA.

**Keywords:** Nephrology, Calcium, Chronic kidney disease, Drug therapy

## Abstract

Dual endothelin-1 (ET-1) and angiotensin II (AngII) receptor antagonism with sparsentan has strong antiproteinuric actions via multiple potential mechanisms that are more pronounced, or additive, compared with current standard of care using angiotensin receptor blockers (ARBs). Considering the many actions of ET-1 and AngII on multiple cell types, this study aimed to determine glomeruloprotective mechanisms of sparsentan compared to the ARB losartan by direct visualization of its effects in the intact kidney in focal segmental glomerulosclerosis (FSGS) using intravital multiphoton microscopy. In both healthy and FSGS models, sparsentan treatment increased afferent/efferent arteriole diameters; increased or preserved blood flow and single-nephron glomerular filtration rate; attenuated acute ET-1 and AngII–induced increases in podocyte calcium; reduced proteinuria; preserved podocyte number; increased both endothelial and renin lineage cells and clones in vasculature, glomeruli, and tubules; restored glomerular endothelial glycocalyx; and attenuated mitochondrial stress and immune cell homing. These effects were either not observed or of smaller magnitude with losartan. The pleiotropic nephroprotective effects of sparsentan included improved hemodynamics, podocyte and endothelial cell functions, and tissue repair. Compared with losartan, sparsentan was more effective in the sustained preservation of kidney structure and function, which underscores the importance of the ET-1 component in FSGS pathogenesis and therapy.

## Abstract 2

This paper reports intravital imaging-based mechanistic studies that identify the glomeruloprotective and tissue remodeling mode of action of the dual endothelin-1 and angiotensin II receptor antagonist sparsentan.

## Graphical abstract

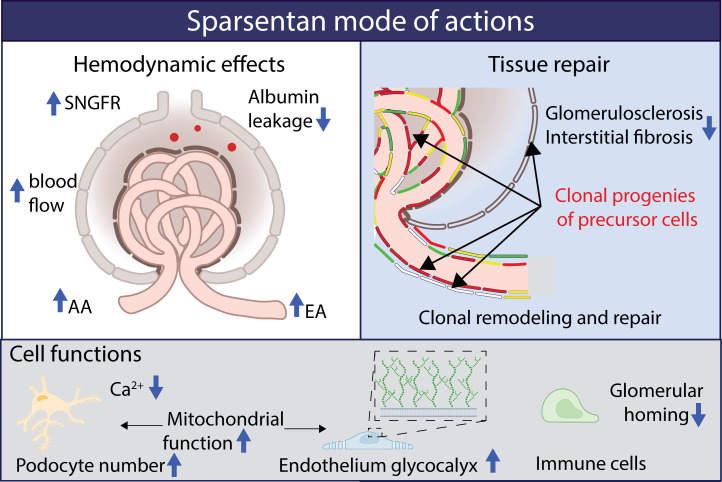

## Introduction

Focal segmental glomerulosclerosis (FSGS) refers to the typical focal and segmental renal histopathology pattern of glomerular scarring that represents a variety of kidney diseases that may result in progressive chronic kidney disease (CKD) and end-stage kidney disease (ESKD). FSGS accounts for about 5% of the approximately 750,000 US adults with ESKD (1). Most primary, secondary, and genetic causes of FSGS are associated with the injury and loss of podocytes, a special cell type of the glomerular filtration barrier (GFB). In cases of genetic FSGS the lesions develop due to defects in various proteins of the podocyte actin cytoskeleton and slit diaphragm complex (2, 3). Recent mechanistic insights based on genetic mutations, multiple animal models, and human clinical studies highlighted the pathogenic role of at least one such protein called transient receptor potential cation channel, subfamily C, member 6 (TRPC6), which mediates podocyte Ca^2+^ influx (4–7). TRPC6 gain-of-function mutations augment angiotensin II–dependent (AngII-dependent) increases in podocyte calcium, causing dysfunction and loss of podocytes and hereditary FSGS (7, 8). A mouse model with approximately 4-fold overexpression of wild-type TRPC6 only in podocytes develops human FSGS-like kidney disease, which has been a useful tool for research (5). In both humans and animal models of glomerulosclerosis, albuminuria is the first sign of glomerular dysfunction, which is often later associated with renal function decline and tissue sclerosis (3). Many patients with FSGS have a progressive course of CKD and ultimately require renal replacement therapy, until new and highly efficacious therapies are developed that can stop or reverse kidney function decline.

Sparsentan is a first-in-class, orally active, single-molecule dual-selective antagonist of the AngII subtype 1 receptor (AT_1_R) and the endothelin A receptor (ET_A_R) (9, 10). Results of recent clinical studies demonstrated strong antiproteinuric actions of sparsentan versus the angiotensin receptor blocker (ARB) irbesartan in patients with FSGS (11–14) and also IgA nephropathy (15), suggesting nephroprotective effects of the drug. Considering the large spectrum of renal actions of AngII and endothelin-1 (ET-1), including vasoconstriction of the afferent and efferent arterioles (AAs and EAs) and mesangial contraction that reduce blood flow and glomerular filtration rate, and their upregulation in kidney injury including FSGS (13), dual inhibition of their actions, as occurs when using sparsentan, is expected to exert multiple actions in many renal cell types and consequently result in a variety of renoprotective mechanisms. Based on recently published and emerging evidence in different models of glomerular disease, sparsentan may preserve glomerular filtration rate and GFB structure and function, inhibit renal cell growth and extracellular matrix production together with antisclerotic, antifibrotic, and antiinflammatory mechanisms, and may augment endogenous tissue repair (13, 16). However, the clear nature of these mechanisms and consequently the primary modes of action of sparsentan, in particular in proteinuric kidney diseases, are not yet completely understood. One major bottleneck of progress in improving our mechanistic understanding of FSGS pathophysiology and therapeutic mechanisms has been the inability to study the structurally and functionally highly complex kidney tissue in its native environment. To advance the field, the present study applied a direct intravital visual approach and comprehensive analysis to study the complexity of sparsentan actions on hemodynamics, cell biology functions, and tissue remodeling.

Recent advances in high-power intravital imaging using multiphoton microscopy (MPM) have made it possible to directly visualize over time the development and progression of glomerular disease processes in the intact mouse kidney in vivo in unprecedented detail (17–23). In addition, transgenic mouse models are available that allow renal cell–specific expression of fluorescent reporters (e.g., the multicolor Confetti construct) (20, 24–26) for genetic cell fate tracking and to study single-cell-based changes in functional (e.g., intracellular calcium) (18, 25, 26) and ultrastructural parameters of the GFB, renal tubules, and microvessels with subcellular resolution in combination with in vivo MPM imaging (22, 25–28). The unique ability of this approach to be able to measure cell calcium in podocytes, AA and EA vascular smooth muscle cells (VSMCs) (18, 25, 26), albumin leakage through the GFB (21, 22), AA/EA/glomerular capillary diameter (19, 29), glomerular endothelial surface layer (19, 21, 23, 30), and immune cell homing (21, 31, 32) has been demonstrated previously.

The present study used intravital MPM imaging of genetically engineered mouse models with fluorescent lineage tags and calcium reporters in various kidney cell types (podocytes, endothelial and vascular progenitor and smooth muscle cells, and tubular epithelia) to quantitatively visualize the mode of action and compare the effects of sparsentan and the ARB losartan on glomerular hemodynamics, GFB function, and kidney tissue remodeling under physiologic conditions and in FSGS.

## Results

### Effects of sparsentan on glomerular hemodynamics under physiological conditions.

**Figure 1:**
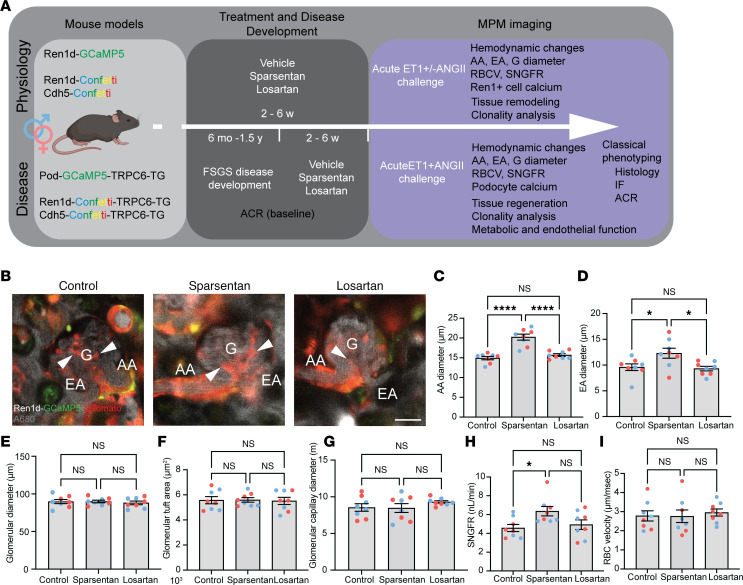
General study design and the effects of sparsentan and losartan on glomerular hemodynamics in the control healthy Ren1d-GCaMP5/tdTomato mouse kidney. (**A**) Schematic illustration of the specific transgenic mouse strains used for either physiological (6–8 weeks old) and FSGS disease models (6 months to 1.5 years old) (left panel), duration of treatment and disease development (center panel), and the applied MPM imaging and classic phenotyping readouts (right panel) in each model. IF, immunofluorescence. (**B**) Intravital MPM images of control healthy Ren1d-GCaMP5/tdTomato mouse kidney glomeruli (G) with their afferent (AA) and efferent (EA) arterioles from no-drug control, sparsentan-, and losartan-treated mice as indicated. The circulating plasma was labeled by i.v. injected albumin–Alexa Fluor 680 (gray). Note the presence of the genetically encoded calcium reporter GCaMP5 (green) and the calcium-insensitive tdTomato (red) in cells of the renin lineage, including AA and EA VSMCs, and extraglomerular and intraglomerular mesangial cells (arrowheads). Scale bar: 20 μm. (**C**–**I**) Statistical summary of the measured hemodynamic parameters (*n* = 8 each), including AA (**C**), EA (**D**), and glomerular diameters (**E**), glomerular tuft area (**F**), glomerular capillary diameter (**G**), single-nephron glomerular filtration rate (SNGFR, **H**), and glomerular capillary red blood cell velocity (**I**). Data represent mean ± SEM. *P < 0.05, ****P < 0.0001 using 1-way ANOVA with Tukey’s multiple-comparison test. For all panels, *n* = 10 measurements averaged for each of the *n* = 8 mice (*n* = 4 males [blue] and *n* = 4 females [red]) in each group.

We first tested the effects of sparsentan as compared with losartan on glomerular hemodynamics in the normal healthy kidney (Figure 1, A–I). Ren1d-GCaMP5/tdTomato mice (6–8 weeks old) that express the calcium reporter GCaMP5 and the non–calcium-sensitive tdTomato in cells of the renin lineage (including AA/EA VSMCs and intraglomerular mesangial cells; Figure 1B) were treated chronically with no-drug control, sparsentan (120 mg/kg body weight), or losartan (10 mg/kg body weight) daily for 6 weeks and prepared for intravital MPM imaging of numerous structural and functional parameters of glomerular hemodynamics at the single-nephron level. Sparsentan (6-week treatment) markedly increased AA (20.23 ± 0.76 vs. 14.94 ± 0.43 μm in control; Figure 1C) and EA diameters (12.30 ± 0.98 vs. 9.59 ± 0.64 μm in control; Figure 1D) and single-nephron glomerular filtration rate (SNGFR) (6.36 ± 0.50 vs. 4.57 ± 0.38 nL/min in control; Figure 1H). No changes were observed in other hemodynamic parameters, including glomerular diameter or tuft area, glomerular capillary diameter, and red blood cell (RBC) velocity (Figure 1, E–I). Losartan did not cause changes in any of these glomerular hemodynamic parameters (Figure 1, C–I).

**Figure 2:**
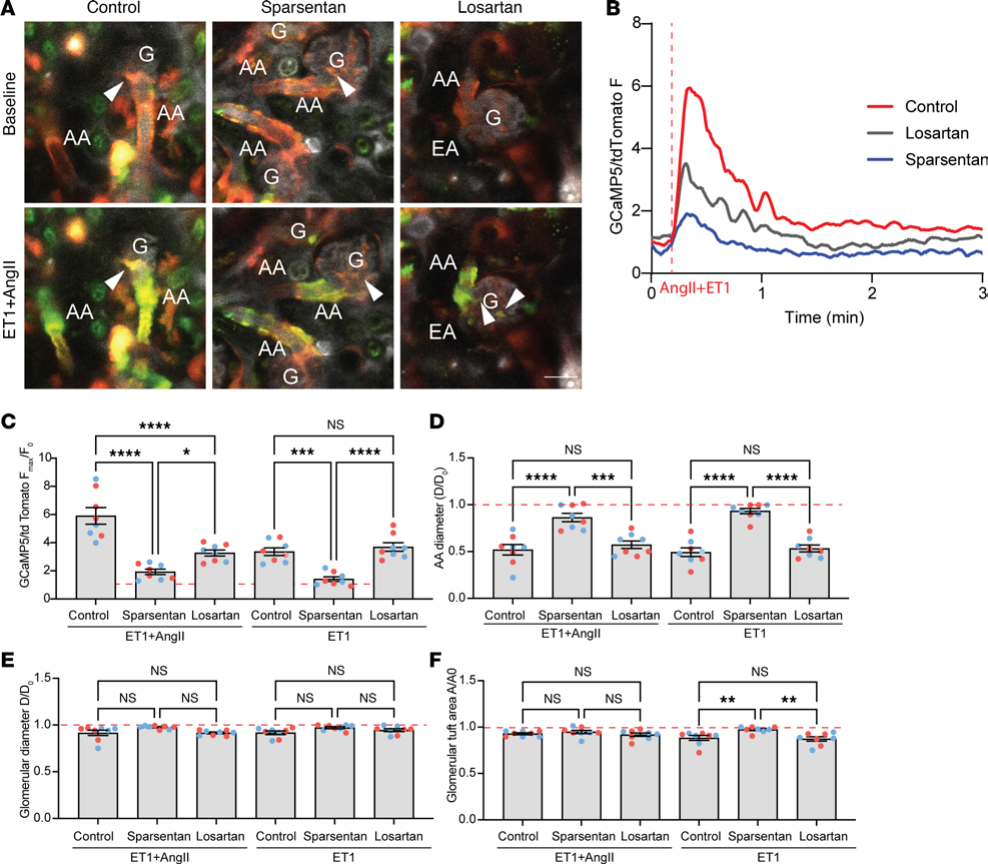
Effects of sparsentan and losartan on glomerular hemodynamic changes caused by acute agonist–induced vasoconstriction in control healthy Ren1d-GCaMP5/tdTomato mice. (**A**) Intravital MPM images of glomeruli (G) with their afferent (AA) and efferent (EA) arterioles before (top panel) and after (bottom panel) the bolus injection of endothelin (ET-1, 50 ng/kg) with AngII (400 ng/kg) combined into the cannulated carotid artery in no-drug control (left), sparsentan-treated (center), and losartan-treated (right) mice. The circulating plasma was labeled by i.v. injected albumin–Alexa Fluor 680 (gray). Note the presence of the genetically encoded calcium reporter GCaMP5 (green) and the calcium-insensitive tdTomato (red) in cells of the renin lineage, including AA and EA VSMCs, and extraglomerular and intraglomerular mesangial cells (arrowheads). Scale bar: 20 μm. (**B**) Time-lapse recordings of AA VSMC calcium (GCaMP5/tdTomato fluorescence [F] ratio) changes in response to bolus ET-1+AngII injection into the carotid artery (vertical line) in the 3 experimental groups. (**C**–**F**) Statistical summary of the measured hemodynamic parameters (*n* = 8 each) normalized to baseline (ratio of maximum effect/before injection), including GCaMP5/tdTomato fluorescence ratio (F_max_/F_0_) in VSMCs of the AA (**C**), and the AA (**D**) and glomerular diameters (**E**), and glomerular tuft area (**F**). Data represent mean ± SEM. *P < 0.05, **P < 0.01, ***P < 0.001, ****P < 0.0001 using 1-way ANOVA with Šidák’s multiple-comparison test. For all panels, *n* = 10 measurements averaged for each of the *n* = 8 mice (*n* = 4 males [blue] and *n* = 4 females [red]) in each group.

In addition, we tested the ability of sparsentan to prevent glomerular hemodynamic changes caused by acute agonist–induced vasoconstriction. ET-1 (50 ng/kg) with or without AngII (400 ng/kg) was injected in bolus into the cannulated carotid artery of the same Ren1d-GCaMP5/tdTomato mice (Figure 2A) that were pretreated with no-drug control, sparsentan (120 mg/kg), or losartan (10 mg/kg) for 6 weeks. In control mice, combined injection of ET-1+AngII caused strong vasoconstriction of the AA (51.93% ± 5.62% of baseline diameter), which was also confirmed by the substantial elevations in AA VSMC calcium (5.90 ± 0.59-fold of baseline; Figure 2, A–D, and Supplemental Video 1; supplemental material available online with this article; https://doi.org/10.1172/jci.insight.177775DS1). The glomerular diameter (91.61% ± 2.80% of baseline diameter) and tuft area (92.79% ± 0.99% of baseline diameter) were similarly reduced (Figure 2E). While losartan inhibited only the elevations in dual agonist–induced AA VSMC calcium (3.27 ± 0.21-fold of baseline; Figure 2, B and C), but not the other hemodynamic parameters (Figure 2, D–F), sparsentan almost completely abolished dual agonist–induced AA VSMC calcium elevations (1.93 ± 0.19-fold of baseline) and AA vasoconstriction (86.41% ± 4.44% of baseline diameter; Figure 2, B–D, and Supplemental Video 1). These effects of sparsentan were more pronounced than those of losartan (Figure 2, B–F). While sparsentan partially attenuated the agonist effects on glomerular diameter and tuft area, these effects did not reach statistical significance (Figure 2, E and F). ET-1 injection alone produced similarly strong AA/glomerular contractions (Figure 2, C–F). While losartan had no effect on any of the glomerular hemodynamic changes (Figure 2, C–F), treatment with sparsentan almost completely abolished ET-1–induced elevations in AA VSMC calcium (1.41 ± 0.17-fold of baseline compared with 3.36 ± 0.27-fold in control), AA vasoconstriction (93.26% ± 2.85% of baseline diameter compared with 49.35% ± 4.62% in control), and the reduction in glomerular tuft area (97.30% ± 1.14% of baseline compared with 88.31% ± 2.58% in control; Figure 2, C–F). In addition to the effects on AA VSMCs, sparsentan almost completely abolished the ET-1–induced elevations in intracellular calcium also in intraglomerular mesangial cells (1.52 ± 0.07-fold of baseline compared with 4.52 ± 0.46-fold in control) and in cells of the distal convoluted tubule (1.17 ± 0.10-fold of baseline compared with 2.71 ± 0.32-fold in control) (Supplemental Video 2). The distal tubule effect of ET-1 was likely mediated via the actions of filtered ET-1 in the tubular fluid acting on ET-1 receptors localized at the apical cell membrane, based on the delayed nature of this effect appearing 30–40 seconds after injection and with a calcium wave propagation pattern from the apical toward the basal cell regions (Supplemental Video 2).

### Effects of sparsentan on kidney tissue remodeling under physiological conditions.

**Figure 3:**
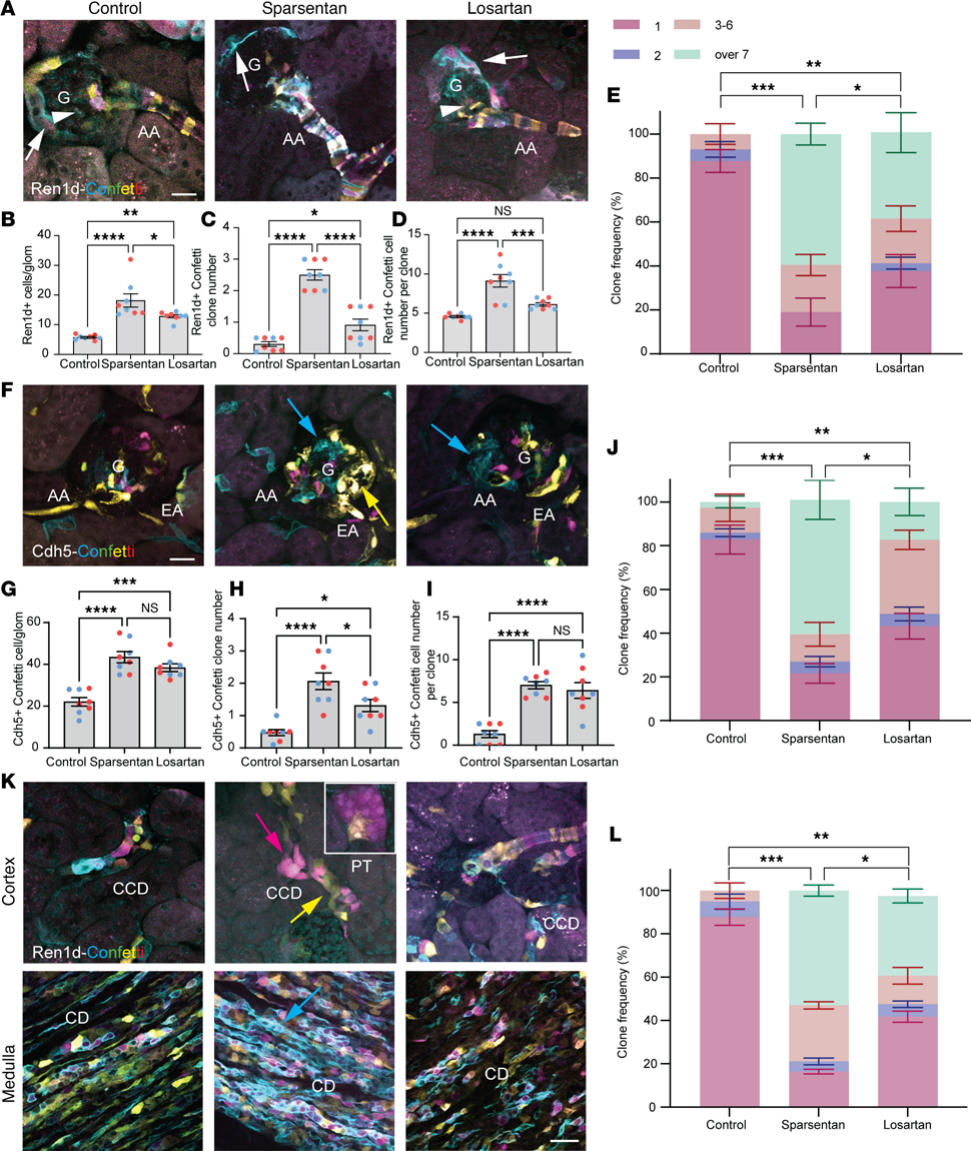
Effects of sparsentan and losartan on glomerular and vascular tissue remodeling in control healthy conditions. (**A** and **F**) Ren1d-Confetti (**A**) and Cdh5-Confetti (**F**) mouse kidney sections from no-drug control (left), sparsentan (center), or losartan (right) treatment. Note the presence of the genetically encoded multicolor Confetti reporter (membrane CFP [blue], nuclear GFP [green], cytosolic YFP [yellow], and RFP [red]) in cells of the renin lineage (including afferent arteriole [AA] vascular smooth muscle and renin cells, extraglomerular and intraglomerular mesangial cells [arrowheads], parietal epithelial cells, and podocytes of the Bowman’s capsule [arrows] in **A**) and in glomerular (**G**), AA, and efferent arteriole (EA) vascular endothelium (**F**). (**B**–**J**) Statistical summary (*n* = 8 each) of the number of Ren1d-Confetti^+^ and Cdh5-Confetti^+^ cells (**B** and **G**), identical color cell groups (clones, blue/yellow arrows in **F**) (**C** and **H**), cells per clone (**D** and **I**), and clone frequency (**E** and **J**) in the various treatment groups. (**K**) Confetti^+^ cortical (top) and medullary (bottom) tubular cells in Ren1d-Confetti mouse kidney sections from no-drug control (left), sparsentan (center), or losartan (right) treatment. Note the presence or absence of clonal (identical color) cell groups in the proximal tubule (PT, inset), cortical (CCD), and medullary collecting duct (CD). Scale bars: 20 μm (all panels). (**L**) Statistical summary (*n* = 8 each) of clone frequency in renal tubules in Ren1d-Confetti mouse kidney sections in the various treatment groups. Data represent mean ± SEM. *P < 0.05; **P < 0.01; ***P < 0.001; ****P < 0.0001 using 1-way ANOVA with Tukey’s multiple-comparison test. NS, not significant. For all panels, *n* = 10 measurements averaged for each of the *n* = 8 mice (*n* = 4 males and *n* = 4 females) in each group.

To test the effects of sparsentan compared with losartan on endogenous kidney tissue remodeling in the normal healthy kidney, Ren1d-Confetti and Cdh5-Confetti mice (6–8 weeks old) were used that express the multicolor Confetti reporter (membrane CFP [blue], nuclear GFP [green], cytosolic YFP [yellow], and RFP [red]) in cells of the renin lineage or endothelium, respectively. Mice were treated with no-drug control, sparsentan (120 mg/kg), or losartan (10 mg/kg) daily for 2 weeks and then prepared for histological analysis of fixed kidney sections. In Ren1d-Confetti mice, sparsentan treatment resulted in a substantial increase in the number of Confetti^+^ cells (18.19 ± 2.23 vs. 5.75 ± 0.28 in control), the number of identical Confetti color cell groups (clones) (2.50 ± 0.16 compared with 0.30 ± 0.07 in control), and the number of individual Confetti^+^ cells per clone (9.13 ± 0.79 compared with 4.56 ± 0.11 in control) in the glomerular tuft, at the glomerular vascular pole and terminal AA segment (Figure 3, A–D). Sparsentan had a similar effect on clonal remodeling of the glomerular and vascular endothelium (Cdh5-Confetti mice; Figure 3, F–J). In summary, sparsentan increased the frequency of larger multicell clones in both the renin and endothelial lineage (Figure 3, E and J). Losartan had a similar effect on both the Ren1d- and Cdh5-Confetti cell populations, but with a reduced magnitude compared with sparsentan (Figure 3, A–J). In addition, various renal cortical and medullary tubule segments, including cells of the proximal tubule, the distal convoluted tubule, and the collecting duct also showed active cellular (clonal) remodeling in response to sparsentan based on the increased frequency of larger multicell clones, and with a modest response to losartan (Figure 3, K and L).

To confirm that sparsentan and losartan administration via custom-made mouse chow in the present study resulted in therapeutically relevant and effective plasma levels, 2 blood samples (early morning and late afternoon consistent with the 12-hour dark/light cycle) were collected from all animals at the end of the 6-week treatment for the measurement of plasma drug levels. Sparsentan and losartan were detectable in the plasma in all mice and time points (data not shown), and there was no difference in systolic blood pressure between the 3 treatment groups in physiological conditions measured by tail cuff (111.2 ± 5.7 mmHg in control, 106.4 ± 2.3 mmHg in sparsentan [*P* = 0.16 vs. control], and 112.2 ± 5.1 mmHg in losartan [*P* = 0.92 vs. control] treatment).

### Effects of sparsentan on glomerular hemodynamics and GFB function in FSGS.

**Figure 4:**
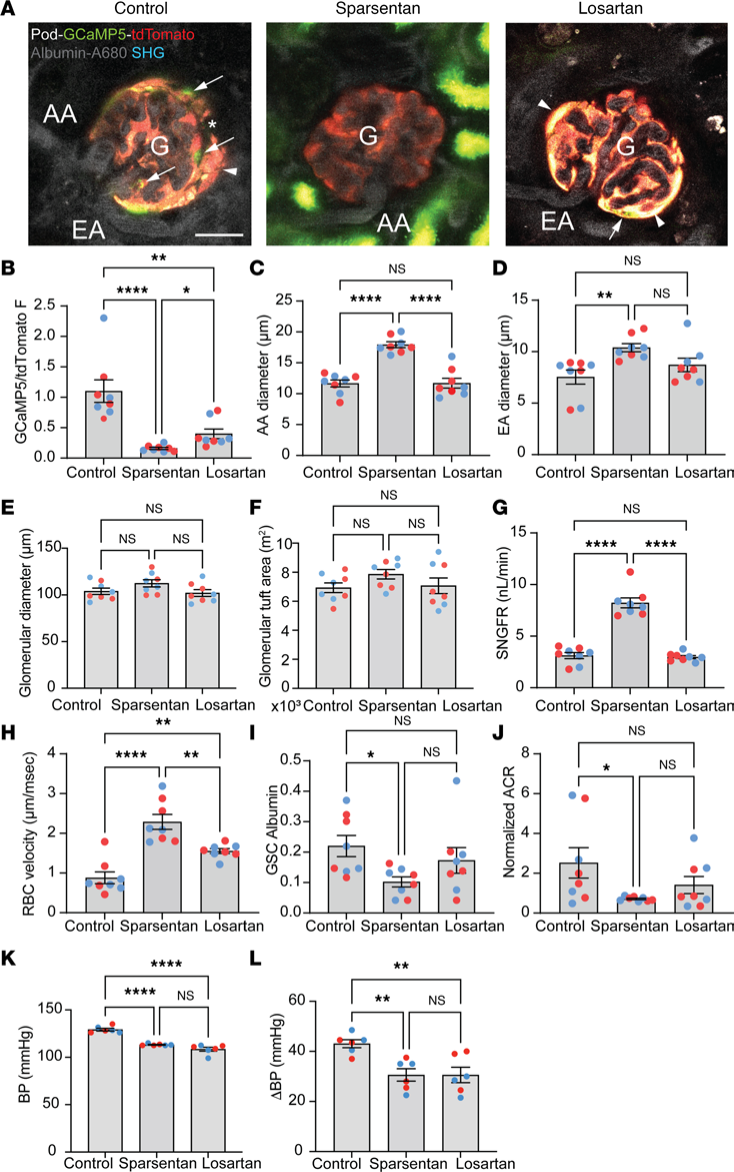
Effects of sparsentan and losartan on glomerular hemodynamics and GFB function in FSGS. (**A**) Intravital MPM images of glomeruli (G) and their afferent (AA) and efferent (EA) arterioles from no-drug control (left), sparsentan-treated (center), and losartan-treated (right) Pod-GCaMP5/tdTomato TRPC6-Tg mice (1.5 years old). The circulating plasma was labeled by i.v. injected albumin–Alexa Fluor 680 (gray). Note the presence of the genetically encoded calcium reporter GCaMP5 (green) and the calcium-insensitive tdTomato (red) in podocytes. Podocytes with high calcium (intense green) are visible in a segmental pattern (arrows) adjacent to areas of adhesions between capillaries and parietal podocytes (arrowheads). Plasma albumin leaked through the GFB is visible in the Bowman’s space (asterisk). Scale bar: 20 μm. (**B**–**J**) Statistical summary of the measured hemodynamic and GFB parameters in the various treatment groups (*n* = 8 each), including podocyte calcium (based on the ratio of GCaMP5/tdTomato fluorescence [F] intensity, **B**), AA (**C**), EA (**D**), and glomerular diameters (**E**), glomerular tuft area (**F**), single-nephron glomerular filtration rate (SNGFR, **G**), glomerular capillary red blood cell velocity (**H**), albumin leakage through the GFB (based on albumin glomerular sieving coefficient [GSC], **I**), and albuminuria (based on urinary albumin/creatinine ratio [ACR] normalized to baseline, **J**). (**K** and **L**) Systolic blood pressure (BP) at baseline (**K**) and elevations (ΔBP) in response to acute AngII injection (**L**) (*n* = 8 each). Data represent mean ± SEM. *P < 0.05; **P < 0.01; ****P < 0.0001 using 1-way ANOVA with Tukey’s multiple-comparison test. NS, not significant. For all panels, *n* = 10 measurements averaged for image-based parameters (panels **B**–**I**) for each of the *n* = 8 mice (*n* = 4 males [blue] and *n* = 4 females [red]) in each group.

We next evaluated the effects of sparsentan as compared with losartan on glomerular hemodynamics under disease conditions using a mouse model of FSGS. Pod-GCaMP5/tdTomato TRPC6-Tg mice were developed that express the calcium reporter GCaMP5 and the non–calcium-sensitive tdTomato only in podocytes, and also feature approximately 4-fold overexpression of TRPC6 in podocytes. These mice (1.5 years old) that are at an advanced age when substantial pathology is present, as reported previously (5), were treated chronically with no-drug control, sparsentan (120 mg/kg), or losartan (10 mg/kg) daily for 6 weeks and prepared for intravital MPM imaging of the same glomerular hemodynamic parameters as before in the physiological model. In contrast with the healthy physiological state and similarly to another preclinical FSGS model using Pod-GCaMP3 mice in our earlier study (18), in no-drug control, Pod-GCaMP5/tdTomato TRPC6-Tg mice had visual signs of ongoing FSGS pathology, including segmental elevations in podocyte calcium, the development of multiple parietal podocytes and adhesions between parietal Bowman’s capsule and glomerular capillary segments, and albumin leakage through the GFB (Figure 4, A–J). Sparsentan treatment markedly improved several parameters of glomerular hemodynamics and GFB function compared with control (Figure 4, B–J), including reductions in podocyte calcium (GCaMP5/tdTomato fluorescence ratio 0.16 ± 0.02 vs. 1.1 ± 0.19 in control; Figure 4B), increased AA (17.92 ± 0.47 vs. 11.65 ± 0.57 μm in control) and EA diameters (10.39 ± 0.40 vs. 7.53 ± 0.69 μm in control; Figure 4, C and D), SNGFR (8.22 ± 0.48 vs 3.11 ± 0.29 nL/min in control; Figure 4G), and glomerular capillary blood flow (RBC velocity 2.29 ± 0.19 vs. 0.88 ± 0.15 μm/ms in control; Figure 4H). In contrast with the low glomerular albumin permeability in control healthy conditions (albumin glomerular sieving coefficient [GSC] below 0.015), as established in previous in vivo MPM imaging studies (33, 34), the presently applied FSGS model featured glomerular albumin leakage (Figure 4I). Importantly, albumin leakage through the GFB (albumin GSC 0.10 ± 0.02 vs. 0.22 ± 0.03 in control; Figure 4I), and the level of albuminuria (urinary albumin/creatinine ratio [ACR] normalized to baseline 0.71 ± 0.04 vs. 2.52 ± 0.76 in control; Figure 4J) also improved markedly in response to sparsentan treatment. Of all the parameters measured, losartan improved only podocyte calcium (Figure 4B) and glomerular capillary blood flow (Figure 4H), but with a lower magnitude compared with sparsentan.

At the end of the chronic treatment for 6 weeks in 6- to 12-month-old TRPC6-Tg (FSGS) mice, systolic blood pressure was measured at the end of the resting (nonfeeding, lowest expected plasma drug levels) phase of their circadian cycle. Direct measurements by pressure transducer via the cannulated carotid artery in anesthetized animals showed equal reduction in blood pressure by sparsentan (113.2 ± 0.4 mmHg) and losartan (108.5 ± 2.0 mmHg) compared with control (129.2 ± 1.4 mmHg) (Figure 4K). To further confirm that the extent of AT_1_R blockade was equal between sparsentan and losartan treatment, systolic blood pressure responses to acute AngII injection were measured via pressure transducer. In control mice, acute bolus injections of AngII (400 ng/kg) caused marked elevations in systolic blood pressure (ΔBP 43.1 ± 1.6 mmHg), which were substantially and equally lowered in mice chronically treated with sparsentan (30.6 ± 2.5 mmHg) or losartan (30.6 ± 3.1 mmHg) (Figure 4L).

**Figure 5:**
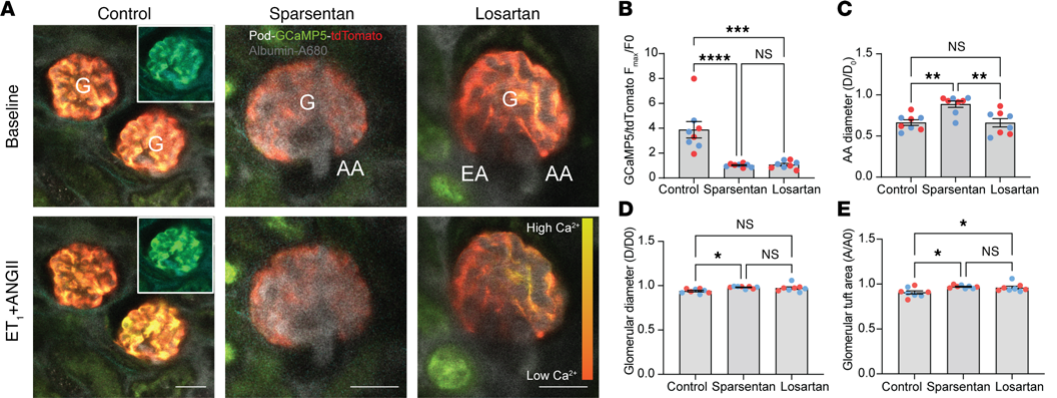
Effects of sparsentan and losartan on changes in glomerular hemodynamics and GFB function caused by acute agonist–induced vasoconstriction in FSGS. (**A**) Intravital MPM images of glomeruli (G) with their afferent (AA) and efferent (EA) arterioles before (top) and after (bottom) the bolus injection of ET-1 (50 ng/kg) and AngII (400 ng/kg) combined into the cannulated carotid artery in no-drug control (left), sparsentan-treated (center), and losartan-treated (right) (for 6 weeks) Pod-GCaMP5/tdTomato TRPC6-Tg mice (1.5 years old). The circulating plasma was labeled by i.v. injected albumin–Alexa Fluor 680 (gray), which also illuminated the AA and EA. Note the presence of the genetically encoded calcium reporter GCaMP5 (green) and the calcium-insensitive tdTomato (red) in podocytes. Insets show the GCaMP5 (green) channel separately to better visualize podocyte calcium changes. Scale bars: 20 μm. (**B**–**E**) Statistical summary of the measured hemodynamic and GFB parameters (*n* = 8 each) normalized to baseline (ratio of maximum effect/before injection), including GCaMP5/tdTomato fluorescence ratio (F_max_/F_0_) in podocytes (**B**), and the AA (**C**) and glomerular diameters (**D**), and glomerular tuft area (**E**). Data represent mean ± SEM. *P < 0.05; **P < 0.01; ***P < 0.001; ****P < 0.0001 using 1-way ANOVA with Tukey’s multiple-comparison test. For all panels, *n* = 10 measurements averaged for each of the *n* = 8 mice (*n* = 4 males [blue] and *n* = 4 females [red]) in each group.

Additional experiments were performed in FSGS mice to test the ability of sparsentan to prevent the changes in glomerular hemodynamics and GFB function caused by acute agonist–induced vasoconstriction, the same way it was tested in the control healthy kidney (Figure 2). ET-1 (50 ng/kg) and AngII (400 ng/kg) were mixed together and injected in as a bolus into the cannulated carotid artery of the same Pod-GCaMP5/tdTomato TRPC6-Tg mice that were pretreated with no-drug control, sparsentan (120 mg/kg), or losartan (10 mg/kg) for 6 weeks. In control mice, ET-1+AngII injections produced major elevations in podocyte calcium (3.88 ± 0.66-fold of baseline), strong vasoconstriction of the AA (66.31% ± 3.4% of baseline diameter), and reductions in glomerular diameter (93.99% ± 0.88% of baseline) and tuft area (90.65% ± 1.85% of baseline) (Figure 5, A–E). While losartan had no effect on most of these parameters except on reducing agonist-induced podocyte calcium elevations (Figure 5B) and glomerular tuft area (Figure 5E), treatment with sparsentan almost completely abolished ET-1+AngII–induced podocyte calcium elevations (1.02 ± 0.05-fold of baseline), AA vasoconstriction (88.67% ± 3.85% of baseline diameter), and reductions in glomerular diameter (98.14% ± 0.48 % of baseline) and glomerular tuft area (97.00% ± 0.62% of baseline; Figure 5, A–E).

### Effects of sparsentan on kidney tissue remodeling in FSGS.

**Figure 6:**
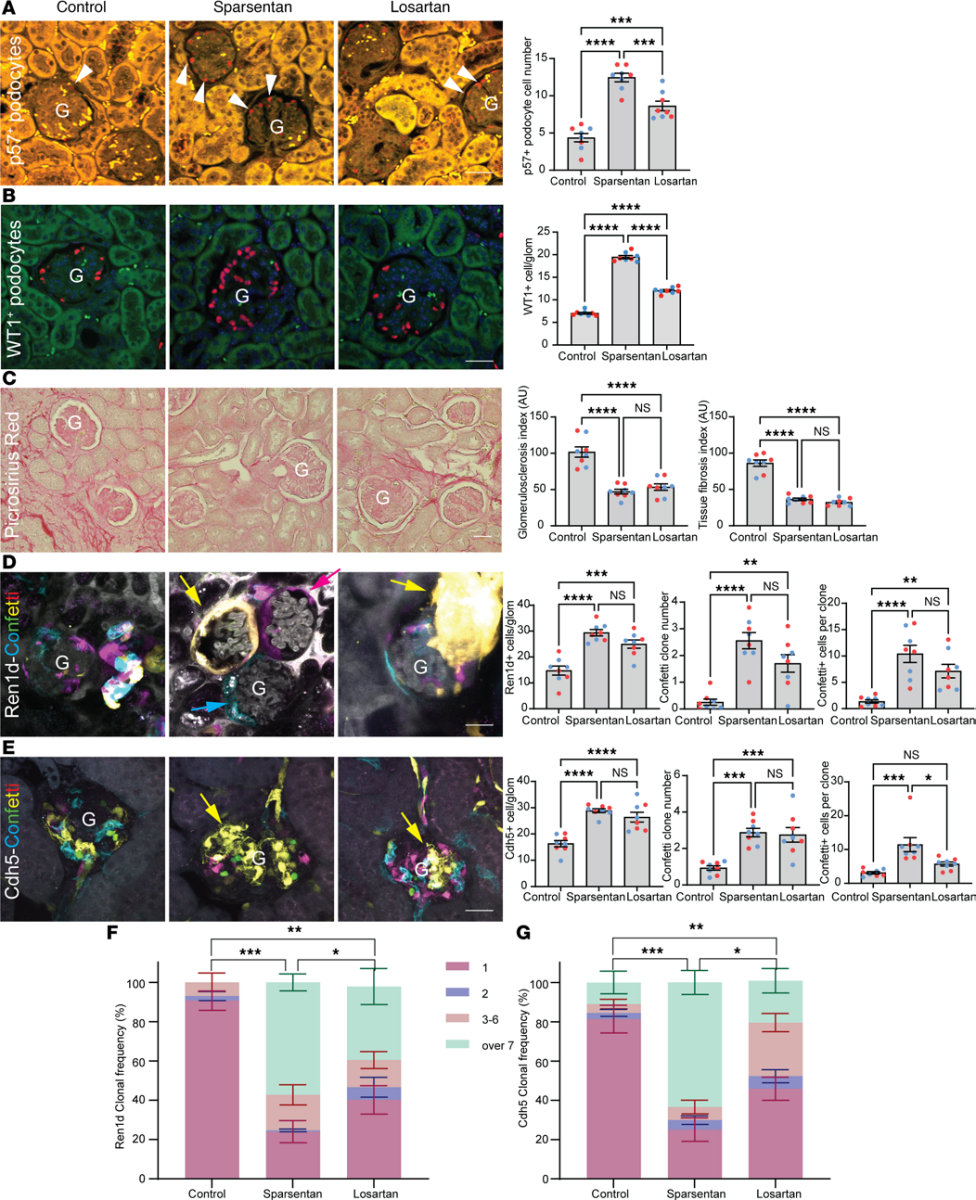
Effects of sparsentan and losartan on podocyte number, glomerulosclerosis and tissue fibrosis, and endogenous tissue regeneration in FSGS. (**A** and **B**) p57 (**A**) or WT1 (**B**) immunolabeling (red, arrowheads) and statistical summary of p57^+^ or WT1^+^ podocyte number/glomerulus in fixed histological sections from no-drug control (left), sparsentan-treated (center), and losartan-treated (right) treated (for 6 weeks) Pod-GCaMP5/tdTomato TRPC6-Tg mouse (1.5 years old) kidneys (*n* = 8 each). Tissue autofluorescence is shown in yellow. (**C**) Picrosirius red (red) staining of kidney sections from the same groups/mice as in **A**, and glomerulosclerosis index (based on Picrosirius red density per glomeruli) and tissue fibrosis index (based on Picrosirius red density per full image frame) in the various treatment groups (*n* = 8 each). (**D**–**G**) Clonal analysis of Ren1d-Confetti (**D**) and Cdh5-Confetti (**E**) Pod-TRPC6-Tg mouse (6 months old) kidneys from same treatment groups as shown in **A** (*n* = 8 each). Note the presence of the genetically encoded multicolor Confetti reporter (CFP [blue], GFP [green], YFP [yellow], and RFP [red]) in cells of the renin lineage (**D**) or vascular endothelium (**E**) and entirely clonal (identical color) glomeruli (yellow/magenta/blue arrows) in sparsentan-treated mice (**C**, center), and to a lower extent in losartan-treated mice (**C**, right). Statistical summary of the number of Confetti^+^ cells, identical color cell groups, cells per clone and clone frequency (**F** and **G**) in the various treatment groups in Ren1d-Confetti (**D** and **F**) and Cdh5-Confetti mice (**E** and **G**). Data represent mean ± SEM. *P < 0.05; **P < 0.01; ***P < 0.001; ****P < 0.0001 using 1-way ANOVA with Tukey’s multiple-comparison test. NS, not significant. For all panels, *n* = 10 measurements averaged for each of the *n* = 8 mice (*n* = 4 males [blue] and *n* = 4 females [red]) in each group. G, glomerulus. Scale bars: 20 μm.

Traditional histology-based phenotyping of podocyte number, glomerulosclerosis, and tissue fibrosis was performed from harvested Pod-GCaMP5/tdTomato TRPC6-Tg mouse kidneys in the 3 treatment groups. Compared with the normally high podocyte number in healthy, non-FSGS mice as established in our recent study using the same protocol (31), podocyte number was low in untreated TRPC6-Tg FSGS mice (Figure 6A). Importantly, the results confirmed the therapeutic benefit of sparsentan on preserving p57^+^ (12.46 ± 0.57 vs. 4.37 ± 0.56 in control; Figure 6A) and WT1^+^ podocyte number (19.46 ± 0.35 vs. 7.02 ± 0.19 in control; Figure 6B) and reducing glomerulosclerosis (47.17 ± 3.20 vs. 101.70 ± 7.15 AU in control) and tissue fibrosis (36.30 ± 1.77 vs. 86.23 ± 4.42 AU in control; Figure 6C). Although losartan also improved these parameters, it was less effective in preserving podocyte number compared with sparsentan (Figure 6, A–C).

To test the effects of sparsentan compared with losartan on endogenous kidney tissue remodeling in FSGS, as described in the physiological condition (Figure 3), Ren1d-Confetti and Cdh5-Confetti mice were crossed with Pod-TRPC6-Tg mice to track the fate of cells of the renin lineage or endothelium, respectively. Pod-TRPC6TG/Ren1d-Confetti and Pod-TRPC6TG/Cdh5-Confetti mice (6 months old) were treated chronically with no-drug control, sparsentan (120 mg/kg), or losartan (10 mg/kg) daily for 6 weeks and then prepared for histological analysis. Sparsentan treatment resulted in substantial increases in the number of Ren1d-Confetti^+^ cells (29.38 ± 1.22 vs. 14.75 ± 1.74 in control), the number of identical Confetti color cell groups (clones) (2.56 ± 0.30 vs. 0.25 ± 0.11 in control), and the number of individual Confetti^+^ cells per clone (10.39 ± 1.63 vs. 1.38 ± 0.32 in control) in glomeruli, including the vascular pole and terminal AA segment (Figure 6D). Sparsentan had a similar effect on clonal remodeling of the glomerular and vascular endothelium in FSGS (Figure 6E). In sum, sparsentan increased the frequency of larger multicell clones in both the renin and endothelial lineage in FSGS (Figure 6, F and G). Entirely clonal (unicolor) glomeruli and arterioles were often observed in response to sparsentan, but not losartan treatment (Figure 6, D and E). Losartan had a similar effect on both the Ren1d- and Cdh5-Confetti cell populations, but with a reduced magnitude compared with sparsentan (Figure 6, D–G).

### Effects of sparsentan on glomerular cell metabolism, endothelial surface layer, and immune cell homing in FSGS.

**Figure 7:**
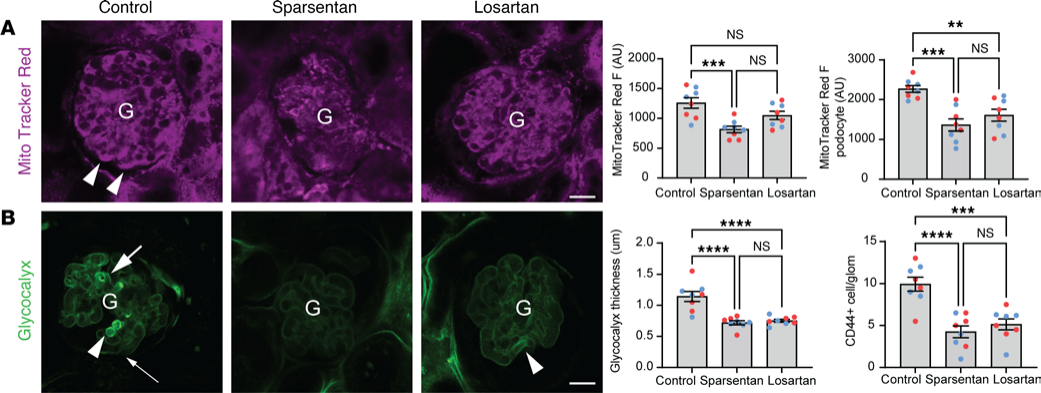
Effects of sparsentan and losartan on glomerular cell metabolism, endothelial surface layer, and immune cell homing in FSGS. (**A** and **B**) Intravital MPM images and quantitative analysis of MitoTracker Red (**A**, magenta) and FITC-WGA (**B**, green) fluorescence in glomeruli (G) from no-drug control (left), sparsentan-treated (center), and losartan-treated (right) (for 6 weeks) Pod-TRPC6-Tg mouse (6 months old) kidneys. Note the intensely labeled podocytes suggesting high level of oxidative stress (**A**, arrowheads) and endothelial glycocalyx heterogeneity (**B**, accumulation in some capillary segments [thick arrows]), but undetectable levels in other regions (thin arrows) in control but not in sparsentan-treated mice. Numerous CD44^+^ (green) immune cells (**B**, arrowheads) were found in the lumen of glomerular capillaries in control FSGS mice. Statistical summary of MitoTracker Red intensity in the entire glomerulus or selectively in podocytes (**A**) and glomerular endothelial surface layer (glycocalyx) thickness and CD44^+^ immune cell homing (**B**) in the various treatment groups (*n* = 8 each). Data represent mean ± SEM. **P < 0.01; ***P < 0.001; ****P < 0.0001 using 1-way ANOVA with Tukey’s multiple-comparison test. NS, not significant. For all panels, *n* = 10 measurements averaged for each of the *n* = 8 mice (*n* = 4 males [blue] and *n* = 4 females [red]) in each group. Scale bars: 20 μm.

To determine specific glomerular functional effects of sparsentan at the cell and molecular level, chronically treated Pod-TRPC6-Tg mice as described above were injected with either MitoTracker Red or FITC-labeled wheat germ agglutinin (FITC-WGA) and anti-CD44–Alexa Fluor 488 antibodies to quantitatively visualize changes in cell metabolism, endothelial surface layer (glycocalyx) density, and glomerular immune cell homing, respectively. Sparsentan treatment resulted in substantial reduction of the mitochondrial membrane potential (an index of oxidative stress) in all glomerular cell types, including podocytes (815.3 ± 52.10 AU vs. 1258 ± 88.96 AU in control; Figure 7A). Sparsentan also improved the glomerular endothelial surface layer compared with the heterogeneous, segmentally high or undetectable levels observed in control (as a sign of endothelial dysfunction; ref. 31) (0.72 ± 0.03 μm vs. 1.14 ± 0.08 μm in control; Figure 7B). In addition, numerous immune cells were observed that had homed to the glomerular capillary lumen in control (as a sign of local inflammation), which were eliminated by sparsentan (4.25 ± 0.70 vs. 9.94 ± 0.84 in control; Figure 7B). Losartan had a similar effect on most, but not all, of these glomerular cell functions compared to sparsentan (Figure 7, A and B).

## Discussion

The present study applied an intravital imaging approach using MPM of the local kidney tissue microenvironment at the single-nephron level to directly and quantitatively visualize the effects of sparsentan in the control healthy kidney and in a disease model of FSGS. The key findings were the broad spectrum and positive classic hemodynamic and tissue regenerative, cell metabolism, and endothelial protective and antiinflammatory effects of sparsentan, and in a number of parameters their superiority in comparison with the ARB losartan. This study represents a major advance toward our improved mechanistic understanding of the specific mode of actions of sparsentan. It also demonstrated the importance of the additive effects or interplay of ET-1 and AngII in the maintenance of renal hemodynamic and glomerular cell biological functions and tissue remodeling in physiological conditions and in the pathogenesis and treatment of FSGS.

In addition to a physiological model, the present study used a disease model: aged mice with podocyte-specific overexpression of wild-type TRPC6, which was shown to result in human FSGS-like disease (5). The human clinical relevance due to pathological features and genetic cause of FSGS due to TRPC6 gain of function makes this mouse model very useful for the preclinical testing of sparsentan. The combination of TRPC6-Tg with cell-specific expression of fluorescent calcium and cell lineage reporters in podocytes, VSMCs, endothelial cells, and mesenchymal progenitor cells was highly valuable in the present study for the direct visualization of the acute or chronic effects of sparsentan or losartan and the actions of ET-1 with or without AngII on these renal cell types. Several molecular and cellular mechanistic details of the mode of action of sparsentan were uncovered in both control healthy and FSGS disease conditions. The results from sparsentan and losartan treatments in physiological models suggest that AngII and ET-1 are active, intrinsic players and important determinants of glomerular hemodynamics and physiological tissue maintenance.

In patients with FSGS, sparsentan lowered proteinuria at 108 weeks of treatment versus irbesartan (14); however, the mechanistic basis for the antiproteinuric effect of sparsentan versus an ARB was not addressed in that study and remains to be elucidated in more detail. As the present MPM imaging approach directly and visually demonstrated (Figures 4–7), a combination of several factors, including improved glomerular hemodynamics, cell biological functional, and tissue remodeling mechanisms, were likely involved in the protective antiproteinuric effects of sparsentan. Importantly, chronic treatment with sparsentan preserved glomerular hemodynamics and GFB functions in FSGS, as well as ameliorated the strong effects of acute vasoconstrictor challenges with the 2 mechanistically and therapeutically relevant agonists (ET-1 and AngII). In contrast with losartan, sparsentan-treated animals demonstrated greater AA/EA diameters and higher SNGFR in physiological as well as FSGS disease conditions (Figures 1 and 4), suggesting that ET-1 is an important contributor to the regulation of glomerular hemodynamics in the healthy kidney and a player in FSGS pathogenesis. Because sparsentan dilated both the AA and EA and increased glomerular capillary RBC velocity (blood flow) considerably (Figure 4H), the increased SNGFR was likely due to an increased filtration coefficient (GFB surface area and permeability) rather than increased glomerular capillary hydrostatic pressure, although this was not measured directly in our study. In addition, increased glomerular blood flow is known to attenuate the rise in oncotic pressure along the glomerular capillary and thereby enhances effective net filtration pressure and SNGFR. The glomerular endothelial remodeling effects (Figures 3 and 6) and the observed trend to increased glomerular tuft and diameter (Figure 4, E and F) with sparsentan treatment are consistent with the above notions. In addition, the reduction in podocyte intracellular calcium levels in response to sparsentan (Figure 4B) is an indication of reduced rather than increased mechanical strain on the GFB, assuming that sparsentan does not directly/indirectly interfere with podocyte calcium handling. The more pronounced effects of sparsentan to abolish acute agonist–induced changes in glomerular hemodynamics (Figure 2) are consistent with the additive protective effect of ET-1 receptor blockade on top of an ARB. Several protective effects of losartan confirmed the well-established pathogenic role of AngII in CKD and FSGS, including the reduced AA VSMC (Figure 2C) and podocyte intracellular calcium levels (Figure 4B), improved glomerular capillary blood flow (Figure 4H), increased podocyte number, and reduced glomerulosclerosis and tissue fibrosis (Figure 6, A–C). The similar effects of sparsentan and losartan on glomerulosclerosis and tissue fibrosis (Figure 6C), despite the clear benefit of sparsentan versus losartan treatment on other parameters such as podocyte number (Figure 6, A and B), suggest that 6 weeks of treatment may not be sufficiently long or the disease stage was too advanced (mice were 1.5 years old) to translate into improved histological readouts in a slowly developing FSGS model such as the applied TRPC6-Tg mouse model. Although it remains to be determined whether the hemodynamic data measured at the single-nephron level translate to global GFR changes, the results strongly suggest that improving renal hemodynamics is one of important modes of action of the therapeutic benefit of dual ET-1 and AngII antagonism with sparsentan. In addition, we cannot exclude that glomerular hemodynamic differences between the sparsentan- and losartan-treated animals are at least in part attributable to better preservation of glomerular structural integrity in the sparsentan treatment group. Interestingly, the present study uncovered the direct effects of ET-1 and sparsentan not only on the AA and EA, but also within the glomerulus on intraglomerular mesangial cells and in numerous renal tubular segments (Figures 1 and 2, and Supplemental Videos 1 and 2). Decreased intracellular calcium in mesangial cells of sparsentan-treated animals is consistent with decreased activation and proliferation seen in the gddY and EIC mouse models of IgA nephropathy (35, 36). These findings are consistent with the expression of the ET_A_R and AT_1_R in these vascular, glomerular, and tubular cell types (37, 38).

Although very few glomerular hemodynamic and GFB parameters were altered by losartan treatment, AT_1_R blockade by losartan inhibited ET-1+AngII–induced elevations in AA VSMC (in physiological model) and podocyte (in FSGS model) calcium (Figure 2C and Figure 4B) and improved glomerular capillary blood flow in the FSGS model (Figure 4H). The observed losartan effect on reducing intracellular calcium, but which was insufficient to cause vasodilation (without changing vascular diameter, Figure 2, C and D), is consistent with the much higher sensitivity of the presently applied MPM calcium imaging approach compared with the classic dimensional readouts (vascular diameter) of AA contractility. The lack of AA vasodilation in response to losartan (Figure 2D) may be explained by the preferential effect of AngII on the EA rather than the AA. In these acute vasoconstrictor injection experiments, the potential changes in EA diameter could not be measured simultaneously due to technical limitations. The increased blood flow without changes in AA/EA diameter in response to losartan (Figure 4, C, D, and H) may be explained by preferential endothelial actions that are supported by the presently observed endothelial protective effects (Figure 7, A and B). These results are consistent with the measured plasma losartan drug levels, which were found to be detectable and in the therapeutic range. However, the more robust and broad effects of sparsentan on many glomerular hemodynamic and GFB parameters (Figures 1, 2, 4, and 6) suggest the primary importance of interplay of ET-1 and AngII signaling in both the physiological maintenance and disease changes in glomerular hemodynamics and GFB functions. It needs to be emphasized that the extent and daily duration of AT_1_R blockade were equal with both sparsentan and losartan treatment. This was indicated by blood pressure data that were similar in the physiological model and equally reduced in FSGS conditions by both sparsentan and losartan compared with control (Figure 4K). Importantly, the acute AngII–induced blood pressure elevations were also equally lowered by both sparsentan and losartan compared with control (Figure 4L). These results further underscore the importance and therapeutic benefit of the ET-1 component of sparsentan and its interplay with AngII signaling in FSGS pathogenesis, independently of blood pressure changes.

The substantial augmentation of protective endogenous tissue remodeling by sparsentan that involves the cells of the renin and endothelial cell lineages (Figures 3 and 6) is an unexpected finding. The renin cell lineage is known to have the ability to function as progenitor cells for parietal epithelial cells and podocytes in glomerular structural and functional repair (39, 40). The results suggest that ET-1 and AngII signaling are major regulators of promoting differentiation rather than self-renewal of renin and endothelial progenitor cells. Based on the superior effects of dual ET-1+Ang II receptor blockade with sparsentan versus inhibition of only AngII receptors with losartan (Figures 3 and 6), ET-1 rather than AngII signaling may be of primary importance in this function. Sparsentan treatment comparably augmented progenitor cell–mediated tissue remodeling in healthy mice (Figure 3) and regeneration in FSGS (Figure 6), suggesting the preservation of its “physiological” tissue remodeling capacity in the disease setting. Importantly, this effect was more pronounced than those of losartan even if renin-angiotensin system inhibition is known to potently increase podocyte derivation from cells of the renin lineage (39). The effects of sparsentan to increase the number of Ren1d-Confetti^+^ clones and the number of cells within 1 clone (Figure 3, C–E) are consistent with its direct effects on progenitor cells and their proliferation. The use of 2 different podocyte markers (p57^+^ and WT1^+^) found preservation of podocyte number by sparsentan and less potently by losartan (Figure 6, A and B) within only a relatively short (6 weeks) treatment compared with the 1.5 years of age and ongoing FSGS pathology development. These results suggests that the recruitment of new podocytes from renin/parietal epithelial cell progenitors (41–43) rather than reduced cell death was involved in this phenomenon. Renin-angiotensin system inhibition and low dietary salt intake that are both associated with increases in the number of renin lineage cells and are known to reduce albuminuria in CKD via glomerular structural improvements independently of blood pressure changes (44). In addition to the renin lineage, the recent development of the Cdh5-Confetti model for genetic fate tracking of endothelial cells at the single-cell level (30, 45) enabled testing of the effect of sparsentan on endothelial precursor cells in the present study. Similarly to renin cells, sparsentan increased the number of Cdh5-Confetti^+^ clones, the number of cells within 1 clone, and the frequency of clones with high cell number (Figure 3, F–J). These results are consistent with the direct effects of sparsentan on endothelial precursor cells and their proliferation. Again, the superior effects of dual ET-1+Ang II receptor blockade compared with the ARB losartan on renal vascular, glomerular, and tubular remodeling and regeneration is an exciting finding. These results strongly suggest that in addition to its effects on renal hemodynamics, kidney tissue regenerative modes of action are involved and are at least as important in the superior therapeutic benefit by sparsentan treatment.

Glomerular disease–relevant actions of both ET-1 and AngII are known to include not only podocyte calcium signaling (Figure 4B and Figure 5B), but also the generation of reactive oxygen species, oxidative stress, inflammation, and degradation of the glomerular endothelial surface layer in models of FSGS (46–50). Therefore, additional in vivo MPM imaging studies were performed to test the effects of sparsentan on these glomerular cell and molecular targets. Similarly to the beneficial effects seen earlier in this study, the results confirmed the therapeutic benefit of sparsentan (in most cases with a trend to be superior to losartan) on normalizing the excessive mitochondrial metabolism (oxidative stress) in podocytes and other glomerular cell types (Figure 7A), the endothelial surface layer in glomerular endothelial cells (Figure 7B), and reducing the glomerular homing of immune cells (Figure 7B). The endothelial glycocalyx protective effects of sparsentan are consistent with recent reports on the similar effects of sparsentan in a different animal disease model or those of ET_A_R inhibition (35, 51). All parameters measured in the present study were tested for the potential impact of sex as a biological variable, and none of the results found differences between males and females (Figures 1–7).

In summary, the present study provides direct in vivo data that point to multiple layers of protective effects of sparsentan in a model of FSGS, demonstrating improvements in glomerular hemodynamics, restoration of the glomerular endothelial surface layer, podocyte calcium signaling, antiinflammatory and metabolic functions, and enhancing endogenous tissue repair. The greater efficacy of sparsentan compared with an ARB in multiple aspects of renal pathophysiology underscores the importance of the interplay of ET-1 and AngII signaling in the pathogenesis and therapy of FSGS.

## Methods

### Sex as a biological variable.

Our study examined male and female animals, and similar findings are reported for both sexes.

### Animals and treatments.

Several transgenic mouse models were used in the present study (Figure 1A), including TRPC6 wild-type (for healthy physiological model) or transgenic mice with podocyte-specific TRPC6 overexpression (Pod-TRPC6-Tg, for FSGS model) that were developed and previously characterized (5). Both models were intercrossed with mice expressing either GCaMP5/tdTomato (the intensely green and highly calcium sensitive fluorescent protein GCaMP5G and the calcium insensitive red fluorescent protein tdTomato) (52) or the multicolor Confetti construct (membrane-targeted CFP, nuclear GFP, cytosolic YFP, or cytosolic RFP) (53) conditionally in either podocytes (Pod) (54), cells of the renin lineage (Ren1d, which include VSMCs, JG renin, mesangial, and parietal epithelial cells) (55), or endothelial cells (Cdh5) (56) using Cre/lox technology as published recently (18, 24, 25, 30, 45). Some of the presently used transgenic mice were provided by academic investigators, such as the Cdh5(PAC)-CreERT2 mice (Ralf Adams, University of Münster, Münster, Germany; Cancer Research UK Scientist via Cancer Research Technology Limited) and the Ren1d-Cre mice (Ariel Gomez, University of Virginia, Charlottesville, Viginia, USA). None of these mice except Pod-TRPC6-Tg showed any morphological or functional abnormalities compared to wild-type mice (not shown). Mouse breeding pairs were purchased from The Jackson Laboratory and were bred to homozygosity for all transgenes and maintained at the University of Southern California in specific pathogen–free quarters according to a homozygous/hemizygous breeding scheme. Tamoxifen was administered by oral gavage (75 mg/kg body weight) once at 4 weeks of age in Cdh5-Confetti mouse models, resulting in endothelial cell–specific expression of reporters (30). Equal numbers of male and female mice at 6–8 weeks of age (healthy physiological model) or at 6 months (Ren1d-Confetti and Cdh5-Confetti TRPC6-Tg FSGS model) or 1.5 years of age (Pod-GCaMP5/tdTomato TRPC6-Tg FSGS model) were used in the present study in 3 separate groups (*n* = 8 each): no-drug control, sparsentan (120 mg/kg body weight), or losartan (10 mg/kg body weight) daily in custom rodent chow for 2 or 6 weeks of follow-up as indicated. Some animals received ET-1 (50 ng/kg body weight) or Ang II (400 ng/kg body weight) or both injected in bolus into the cannulated carotid artery during intravital imaging or blood pressure measurements (described below). At the end of the treatment period, anesthetized mice were perfusion fixed through the heart and the kidneys were harvested for histological analysis.

### Intravital MPM.

Under continuous anesthesia (1%–4% isoflurane inhalant via nose cone), the left kidney was exteriorized through a flank incision and the animals were placed on the stage of an inverted microscope with the exposed kidney placed in a coverslip-bottomed chamber bathed in normal saline, as described previously (20, 57). Body temperature was maintained with a homeothermic blanket system (Harvard Apparatus). Alexa Fluor 680–conjugated bovine serum albumin (Thermo Fisher Scientific) was administered i.v. by retro-orbital injections to label the circulating plasma (30 μL i.v. bolus from 10 μg/mL stock solution). The images were acquired using a Leica SP8 DIVE multiphoton confocal fluorescence imaging system with a 40× Leica water-immersion objective (numerical aperture 1.1) powered by a Chameleon Discovery laser at 960 nm (Coherent) and a DMI8 inverted microscope’s external Leica 4Tune spectral hybrid detectors (emission at 510–530 nm for GCaMP5 and 580–600 nm for tdTomato, and 475–485 nm for detecting second harmonic generation) (Leica Microsystems). When imaging Confetti^+^ cells, CFP, GFP, YFP, and RFP emission was detected at 473, 514, 545, and 585 nm, respectively. The potential toxicity of laser excitation and fluorescence to the cells was minimized by using a low laser power and high scan speeds to keep total laser exposure as low as possible. The usual image acquisition (12-bit, 512 × 512 pixel) consisted of only one *Z*-stack per tissue volume (<1 minute), which resulted in no apparent cell injury. Fluorescence images were collected in time series (xyt, 526 ms per frame) with the Leica LAS X imaging software and using the same instrument settings (laser power, offset, gain of both detector channels). The strong, positive, cell-specific signal (GCaMP5-tdTomato fluorescence) and high-resolution MPM imaging allowed for easy identification of AA and EA VSMCs, mesangial, and tubular cells. Regions of interest (ROIs) were drawn closely over the total cell body of single cells and the changes in the ratio of mean GCaMP5 and tdTomato F/F_0_ fluorescence intensity (ratio was normalized to baseline) were measured in the defined ROI as an index of intracellular calcium changes using the Quantify package of LAS X software (3.6.0.20104, Leica Microsystems).

### Glomerular hemodynamics.

Alexa Fluor 680–conjugated bovine serum albumin was used to label the circulating plasma and the negative labeled (albumin-excluding) RBCs. Quantitative imaging of SNGFR (by measuring the volume/transit time of a systemically injected Lucifer Yellow dye bolus in the early proximal tubule), glomerular diameter, AA and EA diameter was performed as described previously (57, 58). RBC velocity in glomerular capillaries was measured using line (xt) scans of capillary lumen, as previously described (57). GFB function was evaluated based on measurement of albumin leakage into the Bowman’s space (GSC of albumin) (22, 57). ROIs were drawn in glomerular capillary plasma and the Bowman’s space, and image analysis was performed as described previously (23, 34).

### Blood pressure measurements.

Blood pressure was measured by either tail-cuff plethysmography using a Visitech BP-2000 system (Visitech Systems) or directly with a pressure transducer via the cannulated carotid artery in anesthetized animals using an analog single-channel transducer signal conditioner model BP-1 (World Precision Instruments), as described previously (59, 60). All transducer measurement were obtained in late afternoon hours at the end of the inactive, resting (nonfeeding) phase of the animal’s circadian cycle. Tail-cuff measurements were performed in the early morning, and all animals underwent a training period of 5 days before the start of experimental measurements.

### Endothelial surface layer, mitochondrial membrane potential, and immune cell imaging.

FITC-WGA (*Triticum* *vulgaris*; L4895, Sigma-Aldrich), administered via retro-orbital sinus at 2 μg/g body weight, was used to visualize the entire glomerular endothelial glycocalyx. FITC-WGA lectin–positive region of the glomerular endothelial capillary surface was visible immediately after injection. Quantification of glycocalyx thickness was performed on capillary wall line profiles by calculating the width of FITC-WGA signal at half-maximum fluorescence intensity as described previously (19, 23). In cases of capillary segment heterogeneity, areas with the maximum glycocalyx thickness were measured. To quantitatively visualize mitochondrial membrane potential and glomerular immune cell homing, MitoTracker Red (Thermo Fisher Scientific, M7512, dissolved in DMSO) and anti-mouse/human CD44–Alexa Fluor 488 antibodies (BioLegend, 103016) were injected i.v. via retro-orbital sinus, respectively, and imaged as described previously (21, 31, 61).

### Physiologic and biochemical measurements.

Spot urine was collected from animals, and urine albumin was measured by using a murine microalbuminuria ELISA kit (Albuwell M kits, Exocell). Urine creatinine was measured via microplate assay (The Creatinine Companion, Exocell), and the ACR was calculated.

### Tissue processing, immunohistochemistry, and histology.

After anesthesia with a combination of ketamine (100 mg per kg body weight) and xylazine (10 mg per kg body weight), animals were perfused with ice-cold PBS into the left ventricle followed by ice-cold 4% paraformaldehyde (PFA) for 2 minutes each, and tissues were fixed with 4% PFA at 4°C overnight. To visualize Confetti colors, tissues were embedded in OCT after sucrose cryoprotection method (30% sucrose at room temperature for 3 hours) and flash frozen. Cryosections (18 μm thickness) were imaged using the same Leica TCS SP8 microscope as above. Confetti^+^ clonal or unicolor tracing units were defined as numerous directly adjacent individual cells that featured the same Confetti color combination. All 10 possible Confetti color combinations were observed as described previously (30). The counting of Confetti^+^ cells and clones was facilitated by standardized image thresholding using ImageJ (NIH), LAS X software (Leica Microsystems Inc.), and cell-counting algorithms of Imaris 9.2 3D image visualization and analysis software (Bitplane) for imaging same-size *Z*-stacks. Immunofluorescence staining was performed on paraffin sections (6 μm thickness). After antigen retrieval (8 minutes at 95°C in citrate buffer using pressure cooker) and blocking (30 minutes in goat blocking buffer), the sections were incubated with anti-p57 (1:100; ab228635, Abcam) or WT1 (1:100; ab89901, Abcam) primary antibodies followed by incubation with the secondary antibodies conjugated with Alexa 594 (1:500; A-11012, Invitrogen). Slides were mounted by using DAPI-containing mounting media (VectaShield, Vector Laboratories Inc.). Some paraffin sections were stained using Picrosirius red and imaged using the same Leica TCS SP8 microscope as above. Images were analyzed using ImageJ software for measuring Picrosirius red pixel density per unit area as an index of glomerulosclerosis and tubulointerstitial fibrosis.

### Statistics.

Data represent average ± SEM and were analyzed using 1-way ANOVA for multiple groups with post hoc comparison by Tukey’s or Šidák’s test as appropriate. A *P* value of less than 0.05 was considered significant. Statistical analyses were performed using Prism 9.0c (GraphPad Software, Inc.).

### Study approval.

All animal protocols were approved by the Institutional Animal Care and Use Committee at the University of Southern California (Los Angeles, California) and followed the NIH *Guide for the Care and Use of Laboratory Animals* (National Academies Press, 2011).

### Data availability.

All raw data for this manuscript are provided in the supplemental Supporting Data Values XLS file.

## Author contributions

GG, UNS, SD, AI, and JPP conducted the experiments. GG, RK, PWB, and JPP designed the study and wrote the manuscript. All authors approved the final version of the manuscript. Co-author employees of Travere Therapeutics were not involved in data analysis.

## Supplementary Material

Supplemental video 1

Supplemental video 2

Supporting data values

## Version 1 09/03/2024

In-Press Preview

## Version 2 10/08/2024

Electronic publication
